# Effects of *Pelargonium sidoides* root extract on paw oedema, nitric oxide signaling, and synovial vascular changes in a rat model of adjuvant-induced arthritis

**DOI:** 10.1007/s10787-026-02171-z

**Published:** 2026-03-02

**Authors:** Samime Sarli Gunduz, Dikmen Dokmeci, Ozgur Gunduz, Ufuk Usta, Fatma Nesrin Turan

**Affiliations:** 1 Anesthesia Program, Vocational School of Health Services, Trakya University, Edirne, Turkey; 2 Department of Medical Pharmacology, Trakya University Faculty of Medicine, Edirne, Turkey; 3 Department of Pathology, Trakya University Faculty of Medicine, Edirne, Turkey; 4 Department of Biostatistics and Medical Informatics, Trakya University Faculty of Medicine, Edirne, Turkey

**Keywords:** *Pelargonium sidoides*, Rheumatoid arthritis, Nitric oxide, Angiogenesis, Adjuvant-induced arthritis, Synovitis

## Abstract

Rheumatoid arthritis (RA) is characterized by chronic synovitis with pannus formation and progressive joint destruction. Pannus growth relies on angiogenesis, and nitric oxide (NO) contributes to vascular and inflammatory signaling. Although *Pelargonium sidoides* (PS) has not been evaluated in experimental arthritis, prior work suggests that PS can modulate NO production in activated macrophages. Arthritis was induced with complete Freund’s adjuvant (CFA) on day 0. On day 17, rats with ≥ 5% paw swelling were treated. From day 17 to day 27, rats received daily oral *P. sidoides* (100, 200, or 500 mg/kg), ibuprofen (100 mg/kg), or vehicle. Paw diameter was recorded during treatment. On day 27, tissues were collected for histopathology (inflammation, angiogenesis, granuloma formation, synovial hyperplasia) and blood was analysed for nitrite/nitrate (NOx) and oxidative/antioxidant markers (asymmetric dimethylarginine (ADMA), malondialdehyde (MDA), superoxide dismutase (SOD), catalase, and glutathione). All doses of *P. sidoides* reduced paw oedema. The 200 mg/kg dose resulted in NOx levels and synovial microvessel densities that were numerically closer to healthy control values; however, these improvements did not reach statistical significance when compared directly to the vehicle group. Other oxidative markers showed no consistent changes. Plasma glutathione decreased across PS-treated groups without accompanying increases in MDA or body-weight loss, which is compatible with increased utilization during phytochemical clearance rather than overt systemic toxicity. In conclusion, *P. sidoides* improved macroscopic swelling in adjuvant arthritis and, at 200 mg/kg, showed numerical shifts toward control-like values in plasma NOx and synovial microvessel density, although studies with greater statistical power are needed to confirm these exploratory tissue-level signals.

**Supplementary Information:**

The online version contains supplementary material available at 10.1007/s10787-026-02171-z.

## Introduction

Rheumatoid arthritis (RA) is a systemic autoimmune disease characterised by persistent synovitis that progressively damages cartilage and erodes bone. RA affects more than the joints. Cardiovascular and pulmonary involvement is common and contributes to reduced quality of life and increased morbidity (Smolen et al. 2016). Immune-cell activation and pro-inflammatory cytokines remain central to pathogenesis, but oxidative stress appears to add to the disease process. When reactive oxygen species (ROS) increase and antioxidant defences are weakened, inflammatory signalling can be amplified and tissue injury may accelerate. Clinical studies support this view by reporting higher levels of oxidative damage markers, including lipid peroxidation products and protein carbonyls, in patients with RA (Mateen et al. 2016; Quiñonez-Flores et al. 2016; Bilski and Nuszkiewicz 2025; Alka and Mishra 2025).

Pannus formation is another defining feature, characterized as an invasive, vascularized granulation tissue composed of macrophages, fibroblasts, and inflammatory cells that aggressively destroys the adjacent cartilage and bone. As the synovium becomes hyperplastic and invasive, it relies on angiogenesis to maintain nutrient and oxygen supply (Paleolog 2002; Zhao et al. 2025). Nitric oxide (NO) is relevant here because it links vascular regulation with inflammatory signalling. In addition to vasodilation and oedema, NO can promote angiogenic pathways, including those involving vascular endothelial growth factor (VEGF), and may contribute to the abnormal synovial microvasculature seen in RA (Papapetropoulos et al. 1997; Zhao et al. 2025). Although NO production is most pronounced locally within the joint, systemic NOx (nitrite/nitrate) can reflect the circulating nitrosative burden in polyarthritis and is commonly used as an indicator of systemic nitrosative burden in CFA-based arthritis models (Ueki et al. 1996; Mohaddes et al. 2025; Patel et al. 2021). This makes the NO-angiogenesis axis a plausible target for adjunct strategies, while recognising that NO-related effects can be context dependent.

Current pharmacotherapies, particularly disease-modifying antirheumatic drugs (DMARDs), have substantially improved outcomes, yet they largely focus on immune pathways and may not directly address oxidative stress or vascular remodelling (Mueller et al. 2021; Quiñonez-Flores et al. 2016). This gap has sparked interest in adjunct approaches that could influence multiple pathogenic processes in parallel, including inflammation, oxidative burden, and pathological angiogenesis (Zhao et al. 2025; Alka and Mishra 2025).

Within this framework, medicinal plants with established traditional use have attracted renewed attention. *Pelargonium sidoides* DC. (African geranium), indigenous to South Africa, has been used for a wide range of conditions in traditional medicine (Reina et al. 2024). In modern practice, the standardised root extract (EPs® 7630) is widely used for upper respiratory tract infections (Matthys et al. 2003; Lizogub et al. 2007), and has demonstrated anti-inflammatory and antioxidant, alongside antimicrobial and antiviral effects mediated in part through immune modulation (Beil and Kilian 2007; Reina et al. 2024). In vitro studies report that the extract, particularly in combination with lactoferrin, reduces ROS production and nitrite levels in LPS-stimulated macrophages and suppresses interleukin-1β (IL-1β) secretion (Terlizzi et al. 2020). These actions are commonly attributed to its phenolic constituents, including coumarins and proanthocyanidins (Reina et al. 2024; Beil and Kilian 2007).

Given that polyphenols, especially proanthocyanidins, can modulate endothelial function and support vascular integrity (Schini-Kerth et al. 2010), we hypothesised that *P. sidoides* might be associated with favourable changes in processes relevant to pannus-associated inflammation and vascular changes in arthritis, with NO-related pathways as one potential contributor. Accordingly, the present study explored the effects of *P. sidoides* root extract in a rat model of adjuvant-induced arthritis. By integrating biochemical measures with histopathological and vascular outcomes, and focusing on the NO- related vascular and inflammatory, we aimed to investigate the potential association between *P. sidoides* treatment and the modulation of processes relevant to chronic inflammatory joint disease.

## Materials and methods

### Animals and ethics

All experimental procedures were approved by the Trakya University Local Ethics Committee for Animal Experiments (approval date: June 26, 2009; approval no: TÜHADYEK-2004/056) and were conducted in accordance with established ethical guidelines. A total of 48 male Sprague–Dawley rats (8 weeks old, weighing 300–400 g) were used in this study. Animals were housed under standard laboratory conditions (22 ± 1 °C, 55% relative humidity, and a 12-h light/dark cycle) with ad libitum access to standard pellet chow and water.

### Drugs and chemicals

Freund’s complete adjuvant (CFA) was purchased from Sigma-Aldrich (St. Louis, MO, USA). The standardized *P. sidoides* roots root extract (EPs® 7630; Umca® oral solution) was obtained from Abdi İbrahim (Istanbul, Turkey under license from Dr. Willmar Schwabe, Karlsruhe, Germany). Ibuprofen (Dolven®) was obtained from a commercial source (Sanofi S.A., Istanbul, Turkey). Ketamine hydrochloride (Keta-Control®) and xylazine hydrochloride (Xylazinbio® 2%), used for anaesthesia were purchased from Teknovet (Istanbul, Turkey) and Bioveta a.s. (Ivanovice na Hané, Czech Republic), respectively.

### Experimental design

A total of 48 rats were allocated into six groups (*n* = 8 per group): Control, Vehicle, *P. sidoides* root extract at three different doses (100, 200, and 500 mg/kg), and Ibuprofen (100 mg/kg, reference anti-inflammatory control). The vehicle group received a 12% (v/v) ethanol solution to approximate the declared alcohol content of the commercial P. sidoides preparation; other excipients were not separately matched. A separate vehicle-matched control for the commercial ibuprofen formulation was not used. The doses of *P. sidoides* were selected based on allometric scaling from human therapeutic regimens (Food and Drug Administration 2005) and prior experimental studies demonstrating bioactivity in rodent inflammatory models (Nöldner and Schötz 2007; Koch and Biber 2007).

On day-1 (24 h prior to arthritis induction), basal paw thicknesses were measured using a micrometric caliper to establish healthy baseline values for each animal. Following the development of arthritis (induction on day 0), paw measurements were repeated on day 17. Animals that did not exhibit at least a 5% increase in paw thickness compared with baseline values were considered to have insufficient arthritis induction and were excluded from the study according to predefined exclusion criteria. Accordingly, two animals from the Vehicle group and one animal each from the *P. sidoides* 200 mg/kg and ibuprofen groups were excluded.

The treatment protocol was initiated on day 17, to target the established chronic phase of arthritis and continued until day 27. Test substances and vehicle were administered orally by gavage once daily during this period. Doses of *P. sidoides* were calculated based on the declared extract content (820.5 mg extract per mL; manufacturer’s declaration) of the commercial preparation. The effects of treatments on paw oedema were evaluated based on measurements obtained on days 20, 23, and 27, using day 17 values as reference. On the first day of treatment, one animal from the ibuprofen group died due to gavage-related complications and data from this animal were excluded. Final sample sizes for the paw oedema analysis were: control (*n* = 8), vehicle (*n* = 6), *P. sidoides* 100 mg/kg (*n* = 8), *P. sidoides* 200 mg/kg (*n* = 7), *P. sidoides* 500 mg/kg (*n* = 8), and ibuprofen (*n* = 6).

**Fig. 1:**
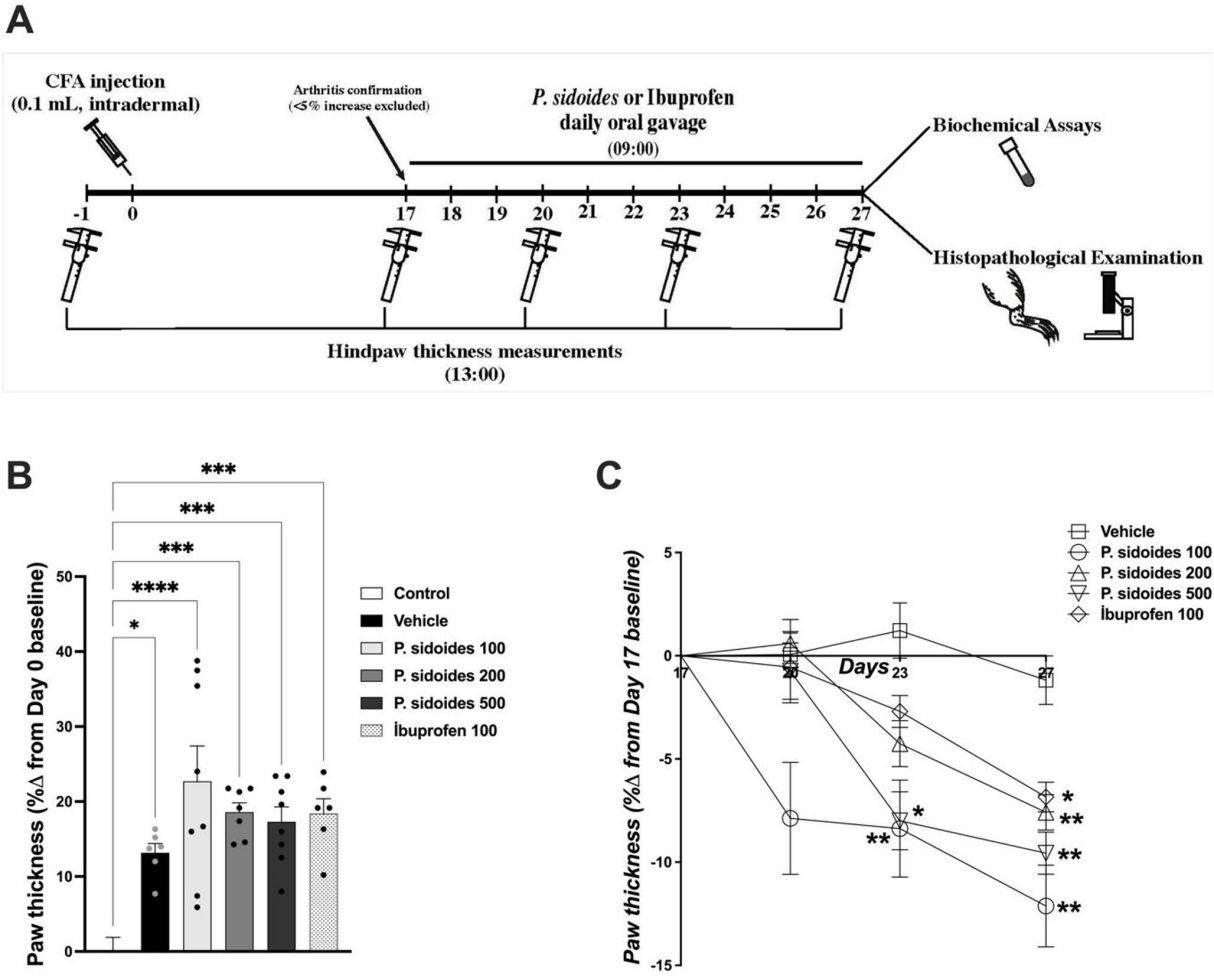
Experimental design and effects of *Pelargonium sidoides* on adjuvant-induced arthritis. **A** Schematic representation of the experimental protocol. Arthritis was induced on day 0, and treatments were administered daily from day 17 to day 27. **B** Evaluation of arthritis induction on day 17 (pre-treatment), expressed as the percentage change in paw thickness relative to the baseline (day-1). All induced groups exhibited significant oedema compared with the control group. **C** Time course of the effects of *P.* *sidoides* (100, 200, and 500 mg/kg) and ibuprofen on established arthritis (days 20, 23, and 27), expressed as percentage change relative to day 17. Data are presented as mean ± SEM (*n* = 6–8). Paw thickness values measured on day 17 were analysed using one-way analysis of variance (ANOVA) followed by Tukey’s post hoc test, whereas changes during the treatment period (days 20, 23, and 27) were analysed using two-way repeated-measures ANOVA followed by Tukey’s post hoc test. *p < 0.05, **p < 0.01, ***p < 0.001, ****p < 0.0001 compared with the control group (**B**) or the vehicle group (**C**)

On day 27, following the completion of the final measurements, rats were anesthetized with a ketamine/xylazine combination (50/10 mg/kg, i.m.) and sacrificed by cardiac exsanguination. Blood samples were collected and centrifuged at 3000 rpm for 10 min at + 4 °C to obtain plasma, which was stored at − 80 °C until biochemical analyses. For histopathological evaluation, tissue samples containing the right and left ankle joints were carefully dissected and preserved under appropriate conditions. The experimental design is summarized in Fig. 1A.

### Induction of adjuvant arthritis

Adjuvant arthritis was induced according to the method originally described by Pearson (Pearson 1956) and later modified by Newbould (Newbould 1963), a standard protocol that remains widely utilized in recent experimental arthritis studies (Mohaddes et al. 2025). Briefly, except for control group, arthritis was induced by a single intradermal injection of 0.1 mL CFA containing heat-killed *Mycobacterium tuberculosis* (10 mg/mL) into the subplantar surface of the right hind paw using a 22-gauge needle.

### Biochemical assays

Plasma oxidative stress parameters were assessed using commercial colorimetric assay kits according to the manufacturers’ protocols. SOD, catalase, and GPx activities, as well as total GSH levels, were measured using kits obtained from Cayman Chemical (Ann Arbor, MI, USA). Plasma MDA levels, as an index of lipid peroxidation, were determined using a commercial assay kit from OxisResearch (Portland, OR, USA). In addition, plasma nitrite and nitrate concentrations (total NOx; stable metabolites of NO**)** were quantified by a spectrophotometric method as previously described by Cortas and Wakid (Cortas and Wakid 1990). Total NOx was used as a systemic nitrosative marker.

### High-performance liquid chromatography analysis

Plasma concentrations of L-arginine, asymmetric dimethylarginine (ADMA), and symmetric dimethylarginine (SDMA) were quantified using high-performance liquid chromatography (HPLC) with fluorescence detection, based on a modified method described by Teerlink et al. (2002).

Chromatographic analyses were performed using a Waters Alliance 2690 XE system coupled to a Model 474 fluorescence detector. Prior to analysis, plasma samples were subjected to solid-phase extraction using Oasis MCX cation-exchange cartridges (Waters). Monomethylarginine was used as the internal standard. Following elution, samples were evaporated to dryness under a nitrogen stream at 60–70 °C, and the residues were derivatized pre-column with ortho-phthaldialdehyde reagent prepared with 3-mercaptopropionic acid.

Separation was achieved on a Symmetry C18 analytical column (3.9 × 150 mm, 5 μm), protected by a Sentry Symmetry C18 guard column. Isocratic elution was performed using a mobile phase consisting of 50 mM potassium phosphate buffer (pH 6.5) containing 8.7% acetonitrile, at a flow rate of 1.1 mL/min. Strongly retained compounds were eluted by flushing the column with a strong solvent (50% acetonitrile) between 20 and 22 min. Fluorescence detection was carried out at excitation and emission wavelengths of 340 and 455 nm, respectively.

*Calibration and linearity:* Calibration curves were generated using nine mixed standard solutions. Method linearity was confirmed over concentration ranges of 1–200 μM for L-arginine and 0.1–20 μM for ADMA and SDMA.

### Histopathological examination

Tissue samples containing the right and left ankle joints were fixed in 10% neutral buffered formalin for 24 h and subsequently decalcified in 10% nitric acid for approximately 36 h. Samples including the metatarsophalangeal joints were processed routinely and embedded in paraffin. Sections of 5 μm thickness were prepared and stained with hematoxylin and eosin (H&E) for histological evaluation under a light microscope (Olympus BX51).

The severity of periarticular inflammation and synovial proliferation was assessed using a semi-quantitative scoring system (0: none, 1: mild, 2: moderate, 3: severe) (Tastekin et al. 2007). Vascular proliferation was evaluated by counting the number of blood vessels in five randomly selected high-power fields (× 40 magnification) and calculating the mean value. In addition, the presence of granuloma formation and oedema was recorded qualitatively.

### Statistical analysis

All analyses and figures were generated using GraphPad Prism v10.6.1 for macOS (GraphPad Software, Boston, MA, USA). Normality was assessed using the Shapiro–Wilk test before selecting parametric or non-parametric tests. Paw thickness on day 17 (expressed as percentage change from the baseline [Day-1]) and biochemical variables were analysed by one-way ANOVA with Tukey–Kramer post hoc test. Changes in paw thickness over the treatment period (days 20, 23 and 27) were analysed by two-way repeated-measures ANOVA (treatment × time), followed by Tukey’s post hoc test; the Geisser–Greenhouse correction was applied when appropriate to account for potential violations of sphericity.

Sample sizes for biochemical assays varied between groups (*n* = 4–8) due to limited plasma volume and the exclusion of hemolyzed samples to ensure data integrity. For GSH, group sizes were: control (*n* = 7), vehicle (*n* = 4), *P. sidoide*s 100 mg/kg (*n* = 5), *P. sidoides* 200 mg/kg (*n* = 7), *P. sidoides* 500 mg/kg (*n* = 8) and ibuprofen (*n* = 5). For catalase, group sizes were: control (*n* = 7) and vehicle (*n* = 5), with other groups ranging from *n* = 7 to *n* = 8. To account for these unequal sample sizes, the Tukey–Kramer method was utilized for post-hoc comparisons, as it calculates critical differences based on the harmonic mean of the group sizes.

Non-parametric outcomes, including histopathological vessel counts, were analysed using the Kruskal–Wallis test with Dunn’s post hoc test. Categorical variables (mononuclear cell infiltration grades and the incidence of granuloma formation or synovial hyperplasia) were compared using Fisher’s exact test; p values were adjusted for multiple comparisons using the Holm–Bonferroni method and are reported as p_adj. All quantitative data, including non-parametric outcomes, are presented as mean ± SEM to facilitate visual comparison. A two-sided *p* value < 0.05 was considered statistically significant.

## Results

### Effect of *P. sidoides* on paw oedema

Injection of CFA into the subplantar surface of the right hind paw induced a pronounced inflammatory response. By day 17 (pre-treatment baseline), paw thickness differed significantly among groups (F(5,37) = 9.87, *p* < 0.0001), with all CFA-injected groups exhibiting increased oedema compared with the control group (Fig. 1B).

Oral *P. sidoides* treatment initiated on day 17 reduced paw oedema over time. In vehicle-treated animals, paw swelling persisted throughout the study. Two-way repeated-measures ANOVA revealed a significant effect of time (F(1.65, 51.04) = 55.90, *p* < 0.0001), a significant effect of treatment (F(4, 31) = 6.11, *p* = 0.0010), and a significant time × treatment interaction (F(6.59, 51.04) = 4.97,* p* = 0.0003), indicating differential temporal responses among the experimental groups (Fig. 1C). All doses of *P. sidoides* significantly reduced paw thickness compared with vehicle during the treatment period. At some time points, oedema reduction was comparable to or numerically greater than that observed with ibuprofen.

Systemic Assessment (Body Weight) Throughout the experimental period, body weight measurements showed no significant decline in *P. sidoides*-treated groups compared to the control group. This stability in body weight indicates that the treatment did not induce systemic toxicity or cachexia associated with severe disease severity (Supplementary Fig. S2).

### Effect of *P. sidoides* on oxidative stress and inflammatory markers

To evaluate the antioxidant and anti-inflammatory effects of *P. sidoides*, biochemical markers were analysed in plasma samples.

**Fig. 2:**
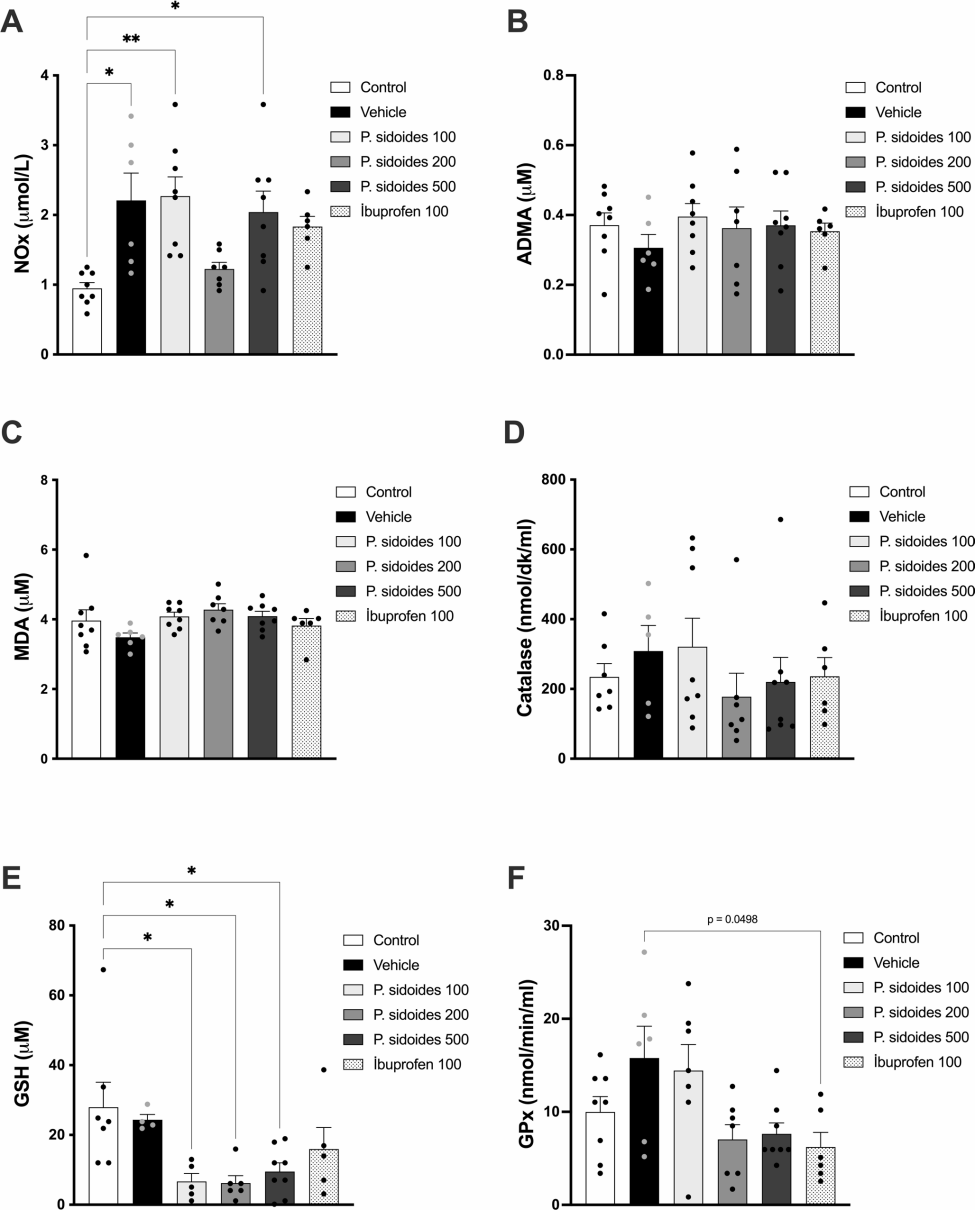
Biochemical evaluation of oxidative stress and inflammatory markers. Effects of *P. sidoides* root extract on (**A**) NO metabolites (NOx; nitrite + nitrate), (**B**) asymmetric dimethylarginine (ADMA), (**C**) malondialdehyde (MDA), (**D**) catalase, (**E**) glutathione (GSH), and (**F**) glutathione peroxidase (GPx) levels in rats with adjuvant-induced arthritis. Data are presented as mean ± SEM. Sample sizes for biochemical assays varied due to technical constraints (per group; individual data points are shown in plots). Specifically, for GSH and catalase, group sizes are detailed in the Statistical Analysis and Results sections. Statistical comparisons were made using one-way ANOVA followed by Tukey’s post hoc test. *p < 0.05, **p < 0.01 versus control

*Nitric oxide metabolites (NOx; nitrite* + *nitrate):* The induction of arthritis led to a marked increase in NOx levels **(**F(5,37) = 5.42, *p* = 0.0008). The Vehicle group, as well as the groups treated with ibuprofen, *P. sidoides* at doses of 100 mg/kg, and 500 mg/kg, exhibited significantly elevated NOx levels compared to the control group (*p* < 0.05). In contrast, the *P. sidoides* 200 mg/kg group was the only treatment group that did not differ significantly from the control group, displaying values numerically closer to the healthy baseline. However, because it also did not differ significantly from vehicle, this pattern was interpreted as an exploratory, control-ward shift rather than a confirmed treatment effect (Fig. 2A).

*Endothelial function*: Plasma concentrations of ADMA, an endogenous inhibitor of nitric oxide synthase, were evaluated to contextualize endothelial NO regulation. ADMA levels did not differ significantly among the study groups (*p* > 0.05; *n* = 6–8) (Fig. 2B), suggesting that the observed NO alterations were not accompanied by parallel changes in circulating ADMA. Similarly, SDMA and L-arginine levels did not differ significantly among the study groups (*p* > 0.05; *n* = 6–8) (Supplementary Fig. S1).

*Lipid peroxidation;* Lipid peroxidation, assessed by MDA, did not differ significantly among the study groups (*p* > 0.05) (Fig. 2C).

*Antioxidant parameters:* Antioxidant enzyme activities and GSH levels were analysed to assess oxidative status. Because of limited plasma volume, sample sizes varied across groups (*n* = 4–8). Catalase activity showed no significant differences among groups (*p* > 0.05; Control *n* = 7, Vehicle *n* = 5, other groups *n* = 7–8) (Fig. 2D).

Plasma GSH levels showed a different pattern. No significant differences were observed among the control, vehicle and ibuprofen groups, indicating that neither arthritis induction nor ibuprofen treatment altered systemic GSH levels. In contrast, all doses of *P. sidoides* were associated with a significant reduction in plasma GSH compared with controls (*p* < 0.05; *n* = 4–8; Fig. 2E).

For GPx, activity did not differ between the control and vehicle groups. However, GPx activity differed significantly between the vehicle and ibuprofen-treated groups (*p* < 0.05; *n* = 6–8), indicating a treatment-related effect of ibuprofen (Fig. 2F).

### Histopathological assessment and angiogenesis

**Fig. 3:**
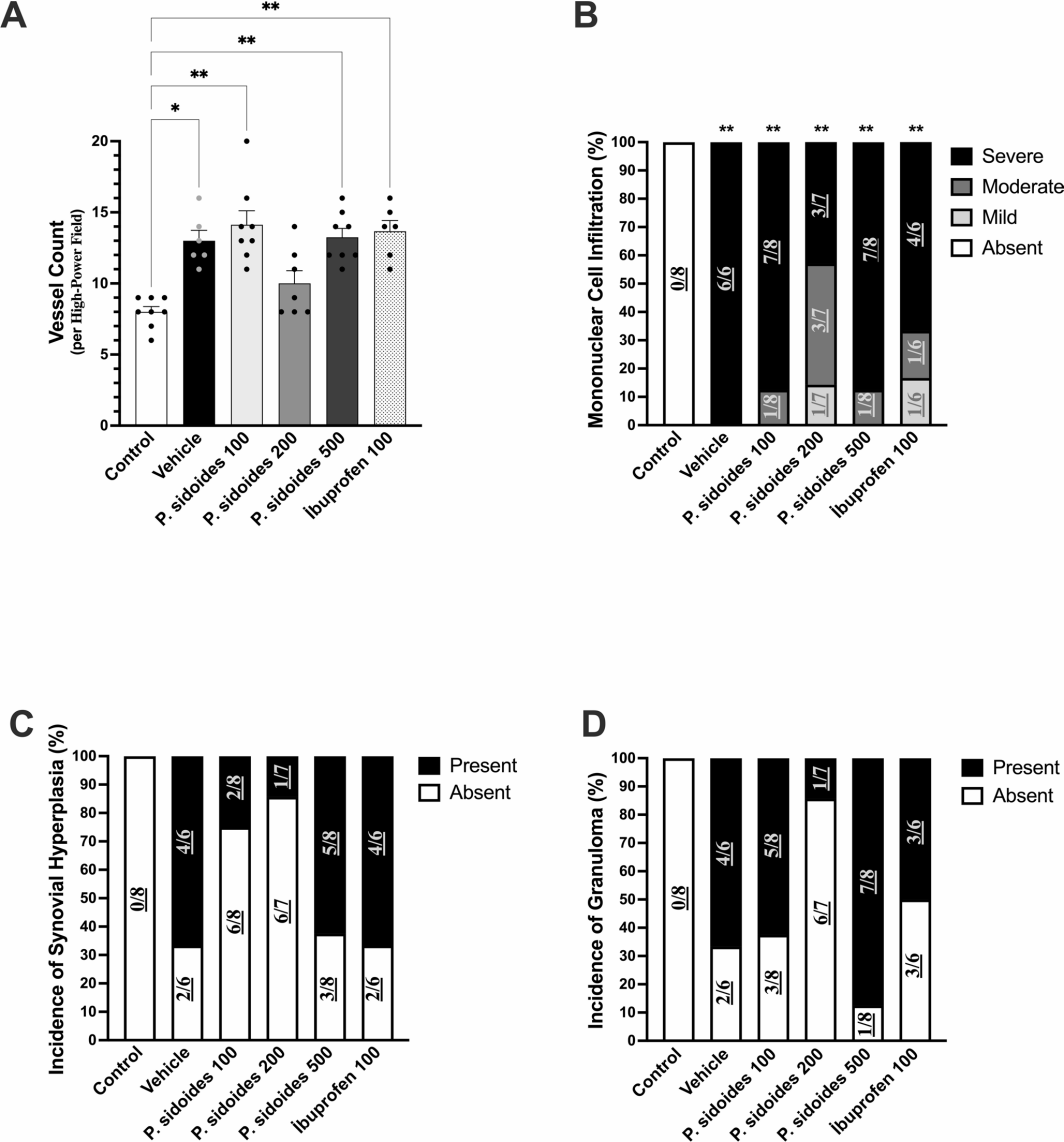
Histopathological assessment of synovial structure and angiogenesis following *P. sidoides* treatment. **A** Vessel count per high-power field (HPF); individual values with mean ± SEM. **B** Mononuclear cell infiltration expressed as percentage distribution of severity grades (absent, mild, moderate, severe). **C** Incidence of synovial hyperplasia (%). **D** Incidence of granuloma formation (%). Numbers within or above bars indicate the ratio of affected animals to the total number analysed in each group (x/n), accounting for exclusions. Vessel counts were analysed by Kruskal–Wallis test with Dunn’s post hoc test. Mononuclear cell infiltration grades and the incidence of synovial hyperplasia and granuloma formation were analysed using Fisher’s exact test with Holm–Bonferroni–adjusted *p* values. *p < 0.05, **p < *0.01 versus control. (n* = *6–8 per group)*

**Fig. 4:**
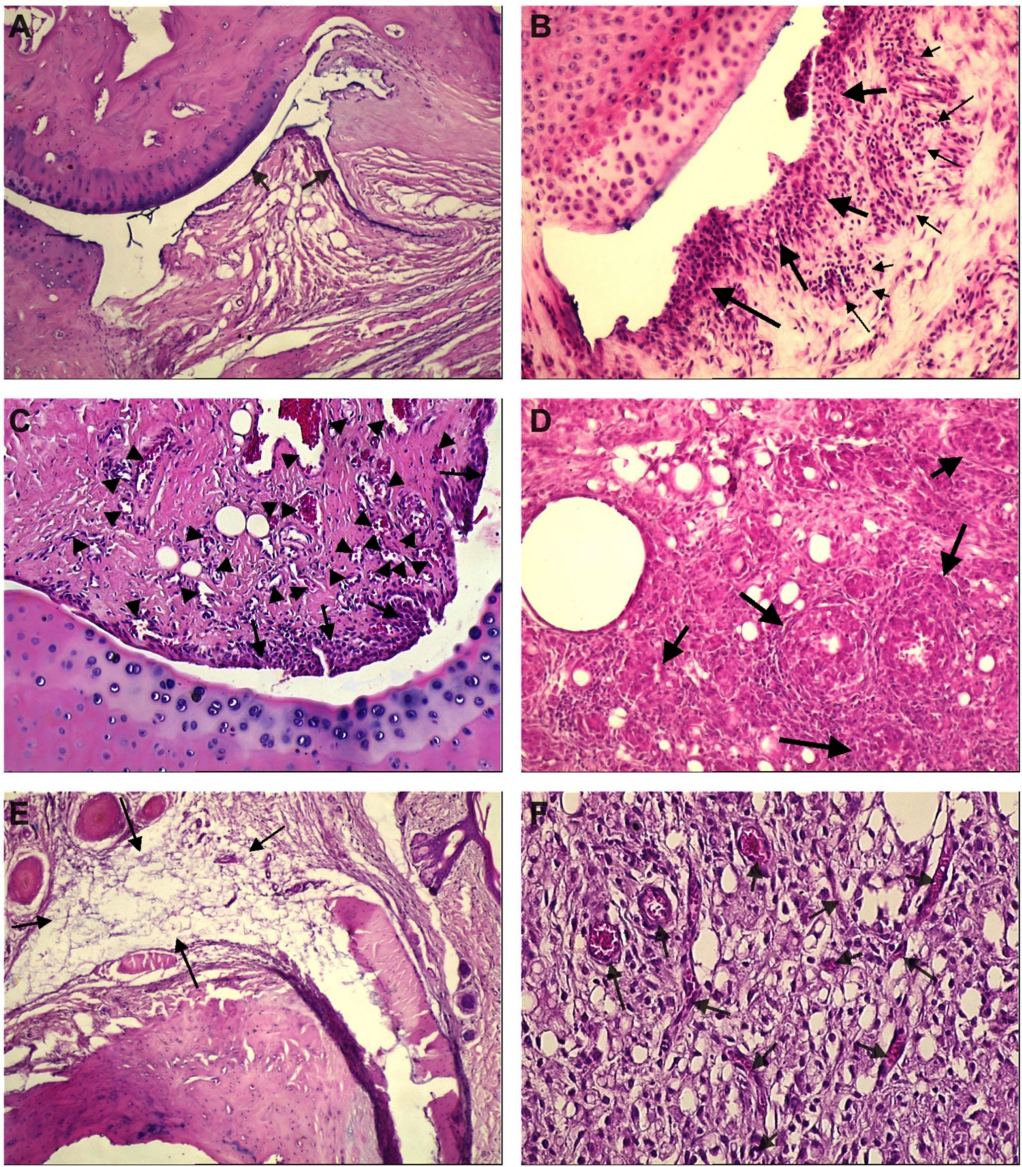
Representative histopathological images of synovial joints (H&E staining). Images are presented at their original magnifications (× 50, × 100, and × 200). **A** Control group showing normal synovial architecture (× 50); arrows indicate the intact synovial lining. **B** Grade 2 synovial inflammation with mononuclear cell infiltration (× 100); large arrows indicate synovial proliferation, small arrows denote inflammatory cell aggregates. **C** Synovial hyperplasia (× 100); arrowheads indicate proliferated microvessels and arrows highlight the expanded synovial layer. **D** Granuloma formation (× 100); arrows indicate granulomatous structures surrounded by inflammatory cells. **E** Oedema within the synovial stroma (× 50); arrows indicate areas of tissue expansion. **F** Pathological angiogenesis with increased vessel density (× 200); arrows indicate multiple vascular profiles

Histopathological evaluation was used to assess treatment-related structural changes (Figs. 3 and 4).

*Angiogenesis (Vessel Count):* Microvessel density differed among groups (Kruskal–Wallis, H(5) = 23.84, *p* = 0.0002). Vessel counts were higher in the vehicle group than in controls (*p* < 0.05; Fig. 3A). The *P. sidoides* 200 mg/kg group was not significantly different from the control group. However, pairwise comparisons versus vehicle did not reach statistical significance for any treatment group (p > 0.05), indicating that the 200 mg/kg group showed an exploratory shift toward control-like values without statistically significant separation from vehicle.

*Mononuclear cell infiltration:* Inflammation was absent in the control group (100% “Absent”), whereas the vehicle group showed severe infiltration in all animals (100% “Severe”). All treated groups remained significantly different from controls (*p* < 0.01; Fig. 3B). Although infiltration persisted, the distribution shifted numerically towards lower severity grades (Moderate/Mild), most notably in the 200 mg/kg group.

*Synovial Hyperplasia and Granuloma:* After adjustment for multiple comparisons, group differences in synovial hyperplasia and granuloma formation did not reach statistical significance (*p* = 0.075 and *p* = 0.06, respectively). Neither endpoint was observed in controls (0%). In the vehicle group, synovial hyperplasia and granuloma formation were each present in 66.7% of animals (4/6). In the *P. sidoides* 200 mg/kg group, the observed incidence was lower (14.3%; 1/7), whereas it remained at 50% in the ibuprofen group (Fig. 3C–D).

## Discussion

We investigated the effects of *P. sidoides* root extract in CFA-induced arthritis, with a focus on NO-related angiogenic changes linked to pannus persistence and tissue remodelling. The CFA model reliably reproduces key features of rheumatoid arthritis, including chronic inflammation and pannus formation (Bendele 2001; Choudhary et al. 2018). In this study, the marked oedema and histopathological changes in the vehicle group confirmed successful model induction and were consistent with our previous findings (Tastekin et al. 2007).

Both *P. sidoides* (100–500 mg/kg) and ibuprofen reduced paw oedema compared with vehicle, indicating comparable macroscopic anti-oedematous activity. This anti-inflammatory response is consistent with recent evidence from the CFA model demonstrating the efficacy of plant-derived fractions in reducing limb swelling and joint thickness (Kaundal et al. 2025). Beyond this shared effect, the 200 mg/kg dose of *P. sidoides* showed the most favourable tissue profile: vascular and synovial measures tended to move towards control values. NOx and microvessel density were not significantly reduced versus vehicle; rather, the 200 mg/kg group was the only group that was no longer statistically different from controls. Because this group was also not significantly different from vehicle, these findings should be interpreted as an exploratory shift toward control-like values rather than evidence of vascular “normalisation” or a definitive suppressive effect. By contrast, ibuprofen reduced oedema and inflammatory infiltration but showed limited effects on indices of joint remodelling and vascular proliferation, in line with the recognised limitation of NSAIDs in altering the mechanisms that drive pannus formation and progressive joint damage (Crofford 2013).

The dose range (100–500 mg/kg) encompasses the human equivalent therapeutic dose (FDA 2005). The observed dose–response pattern was not linear. Although oedema was reduced at all doses, the 100 mg/kg group showed the largest macroscopic improvement, whereas tissue-level measures did not parallel this effect. Instead, NOx and vessel counts followed a U-shaped pattern across the dose range, with the most favourable trends at 200 mg/kg and values closer to vehicle at 100 and 500 mg/kg. This dissociation between macroscopic and microscopic outcomes may be compatible with a hormetic profile, in which benefit is confined to an intermediate dose range. The apparent loss of favourable tissue trends at 500 mg/kg is also consistent with the “double-edged sword” behaviour described for polyphenols, where higher doses may engage counter-regulatory or pro-oxidant mechanisms that offset benefit (Bouayed and Bohn 2010; Calabrese and Mattson 2017). Such dose-dependent effects are plausible given the coumarin and polyphenol constituents of *P. sidoides* (e.g., umckalin, scopoletin) and their links to oxidative pathway modulation (Reina et al. 2024; Terlizzi et al. 2020).

Biochemically, the most prominent signal involved NO-related measures rather than broad suppression of oxidative damage. NOx was elevated in the vehicle group, consistent with the systemic inflammatory nature of the CFA model. While nitric oxide production in arthritis is most pronounced within the joint cavity, often resulting in synovial fluid concentrations that exceed serum levels (Farrell et al. 1992; Ueki et al. 1996), the systemic nature of the disease leads to a measurable spillover of inflammatory mediators into the circulation. Importantly, serum NOx levels have been demonstrated to correlate significantly with clinical disease activity markers (Ueki et al. 1996). Consequently, plasma NOx quantification is commonly used to monitor systemic nitrosative/oxidative burden in the CFA-induced polyarthritis model (Sukketsiri et al. 2016; Mohaddes et al. 2025; Patel et al. 2021). Accordingly, NOx is interpreted here as a systemic surrogate rather than a direct readout of intra-articular NO production. Although none of the treatments produced a statistically significant reduction versus vehicle, the 200 mg/kg dose yielded NOx values numerically closer to control levels. In contrast, MDA and other oxidative stress-related readouts did not demonstrate a consistent, dose-dependent extract-related pattern, which does not support a strong generalized systemic antioxidant effect. The observed decrease in plasma GSH across *P. sidoides*-treated groups may reflect physiological utilization during glutathione-dependent conjugation of coumarins and polyphenols present in the extract (Ketterer 1988; Allocati et al. 2018). However, since glutathione S-transferase activity was not measured, this remains a plausible hypothesis rather than a confirmed mechanism. Importantly, this reduction was not accompanied by increases in lipid peroxidation (MDA) or by body-weight loss, arguing against pathological oxidative depletion or overt systemic toxicity. Xenobiotic biotransformation, including that of coumarins and polyphenols present in *P. sidoides*, can involve glutathione-dependent conjugation pathways; however, because glutathione S-transferase related activity was not assessed here, this interpretation remains plausible rather than mechanistically confirmed. In contrast to recent reports where other herbal extracts restored antioxidant enzyme levels in arthritic rats (Kaundal et al. 2025), the systemic decline in GSH observed in our study may reflect a specific metabolic utilisation associated with the detoxification of *P. sidoides* constituents. Overall, the NOx pattern is consistent with the possibility that the relatively favourable vascular and histological trends observed at 200 mg/kg may be associated with modulation of NO-related inflammatory signaling.

The clearest distinction between *P. sidoides* and ibuprofen emerged in histopathology. Although ibuprofen reduced oedema, synovial hyperplasia and granuloma formation remained relatively frequent (50%), consistent with symptomatic benefit without clear impact on remodelling (Crofford 2013). By comparison, *P. sidoides* at 200 mg/kg produced a marked numerical reduction (66.7–14.3%). Although the reduction did not reach statistical significance after rigorous adjustment (*p* = 0.075 for hyperplasia; *p* = 0.060 for granuloma), the marked numerical difference points to a potential protective effect that merits further evaluation in larger cohorts. While DMARDs are the standard for arresting structural damage, NSAIDs remain the primary first-line therapy for symptomatic relief. Our objective was to evaluate whether *P. sidoides* offers tissue-level benefits distinct from standard cyclooxygenase inhibition, rather than to benchmark it against potent immunosuppressives.

Angiogenesis is a key enabling step in pannus development (Paleolog 2002; Zhao et al. 2025; Wang et al. 2021) and microvessel density increased markedly in vehicle-treated animals. Ibuprofen and *P. sidoides* at 100 and 500 mg/kg did not prevent this increase. The 200 mg/kg dose was distinct: vessel counts were no longer significantly different from controls, although not significantly reduced versus vehicle, supporting a partial normalisation rather than a strong anti-angiogenic effect. These observations align with the established role of angiogenesis in synovial hyperplasia and pannus formation in the CFA model (Paleolog 2002; Zhao et al. 2025; Wang et al. 2021) and are consistent with reports showing that certain plant-derived fractions can attenuate joint inflammation and histopathological changes (Kaundal et al. 2025). Furthermore, the ability of plant-derived constituents to interfere with the signaling pathways that drive pathological neovascularization underscores their potential as adjunct therapies in preventing joint destruction (Alka and Mishra 2025; Wang et al. 2021; Zhao et al. 2025). The parallel shift of NOx and microvessel measures towards control-like values at 200 mg/kg may be consistent with NO-related modulation as one potential mechanistic link (Mateen et al. 2016; Farrell et al. 1992; Ueki et al. 1996).

Several limitations of the current study warrant consideration. First, the absence of specific cytokine and signaling molecule measurements (e.g., TNF-α, IL-1β, IL-6, NF-kB) limits a comprehensive mechanistic evaluation. Second, the use of male rats only precludes the assessment of potential sex-specific pharmacological responses. Furthermore, due to technical challenges in obtaining sufficient volumes of undiluted synovial fluid from rats, plasma biomarkers were used as indicators of systemic inflammatory burden. Moreover, sample sizes for biochemical assays varied due to limited plasma volumes, which may limit the statistical power for certain endpoints. Finally, while the current study focused on a 10-day curative intervention in established arthritis, the limited exposure duration may have reduced sensitivity for detecting tissue-level and vascular remodelling endpoints, and these findings should therefore be considered exploratory. Future research utilizing earlier treatment initiation (e.g., Day 12–14), extended treatment durations, and larger confirmatory cohorts is needed to strengthen mechanistic interpretation and assess durability and long-term safety.

In conclusion, *P. sidoides* reduced paw oedema in CFA-induced arthritis. At 200 mg/kg, it was associated with control-like shifts in NOx and a more favourable vascular/structural histopathological profile than ibuprofen. While these results do not demonstrate definitive disease modification, they support further evaluation of *P. sidoides* as a potential adjunct strategy in rheumatoid arthritis.

## Supplementary Information

Supplementary Material 1.

## Data Availability

Data will be made available on request.

## References

[CR1] Alka MA (2025) Targeting rheumatoid arthritis risk factors with phytochemicals: an anti-inflammatory perspective. Inflammopharmacology 33(7):3561–3582. 10.1007/s10787-025-01830-x 40591213 10.1007/s10787-025-01830-x

[CR2] Allocati N, Masulli M, Di Ilio C, Federici L (2018) Glutathione transferases: substrates, inihibitors and pro-drugs in cancer and neurodegenerative diseases. Oncogenesis 7(1):8. 10.1038/s41389-017-0025-3 29362397 10.1038/s41389-017-0025-3 PMC5833873

[CR3] Beil W, Kilian P (2007) EPs® 7630, an extract from *Pelargonium sidoides* roots inhibits adherence of *Helicobacter pylori* to gastric epithelial cells. Phytomedicine 14:5–8. 10.1016/j.phymed.2006.11.024 17188478 10.1016/j.phymed.2006.11.024

[CR4] Bendele A (2001) Animal models of rheumatoid arthritis. J Musculoskelet Neuronal Interact 1(4):377–385 15758488

[CR5] Bilski R, Nuszkiewicz J (2025) Antioxidant therapies as emerging adjuncts in rheumatoid arthritis: targeting oxidative stress to enhance treatment outcomes. Int J Mol Sci. 10.3390/ijms26072873 40243461 10.3390/ijms26072873 PMC11989177

[CR6] Bouayed J, Bohn T (2010) Exogenous antioxidants–double-edged swords in cellular redox state: health beneficial effects at physiologic doses versus deleterious effects at high doses. Oxid Med Cell Longev 3(4):228–237. 10.4161/oxim.3.4.12858 20972369 10.4161/oxim.3.4.12858 PMC2952083

[CR7] Calabrese EJ, Mattson MP (2017) How does hormesis impact biology, toxicology, and medicine? NPJ Aging Mech Dis. 10.1038/s41514-017-0013-z 28944077 10.1038/s41514-017-0013-z PMC5601424

[CR8] Choudhary N, Bhatt LK, Prabhavalkar KS (2018) Experimental animal models for rheumatoid arthritis. Immunopharmacol Immunotoxicol 40(3):193–200. 10.1080/08923973.2018.1434793 29433367 10.1080/08923973.2018.1434793

[CR9] Cortas NK, Wakid NW (1990) Determination of inorganic nitrate in serum and urine by a kinetic cadmium-reduction method. Clin Chem 36(8 Pt 1):1440–1443 2387039

[CR10] Crofford LJ (2013) Use of NSAIDs in treating patients with arthritis. Arthritis Res Ther 15(3):S2. 10.1186/ar4174 24267197 10.1186/ar4174 PMC3891482

[CR11] Farrell AJ, Blake DR, Palmer RM, Moncada S (1992) Increased concentrations of nitrite in synovial fluid and serum samples suggest increased nitric oxide synthesis in rheumatic diseases. Ann Rheum Dis 51(11):1219–1222. 10.1136/ard.51.11.1219 1466599 10.1136/ard.51.11.1219 PMC1012459

[CR12] Food and Drug Administration (2005) Guidance for industry: estimating the maximum safe starting dose in initial clinical trials for therapeutics in adult healthy volunteers. U.S. Department of Health and Human Services, Rockville, MD

[CR13] Kaundal P, Kamboj A, Malhotra H, Kaushik P (2025) Evaluation of the oxidative stress reduction and anti-inflammatory efficacy of fractions of the methanolic extract of the leaf part of *Ziziphus nummularia* using complete Freund’s adjuvant-induced arthritis in rats. Inflammopharmacology 33(12):7427–7443. 10.1007/s10787-025-01943-3 41217590 10.1007/s10787-025-01943-3

[CR14] Ketterer B (1988) Protective role of glutathione and glutathione transferases in mutagenesis and carcinogenesis. Mutat Res 202(2):343–361. 10.1016/0027-5107(88)90197-2 3057366 10.1016/0027-5107(88)90197-2

[CR15] Koch E, Biber A (2007) Treatment of rats with the *Pelargonium sidoides* extract EPs 7630 has no effect on blood coagulation parameters or on the pharmacokinetics of warfarin. Phytomedicine 14(Suppl 6):40–45. 10.1016/j.phymed.2006.11.026 17188479 10.1016/j.phymed.2006.11.026

[CR16] Lizogub VG, Riley DS, Heger M (2007) Efficacy of a *Pelargonium sidoides* preparation in patients with the common cold: a randomized, double blind, placebo-controlled clinical trial. EXPLORE 3(6):573–584. 10.1016/j.explore.2007.09.004 18005909 10.1016/j.explore.2007.09.004

[CR17] Mateen S, Moin S, Khan AQ, Zafar A, Fatima N (2016) Increased reactive oxygen species formation and oxidative stress in rheumatoid arthritis. PLoS ONE 11(4):e0152925. 10.1371/journal.pone.0152925 27043143 10.1371/journal.pone.0152925 PMC4820274

[CR18] Matthys H, Eisebitt R, Seith B, Heger M (2003) Efficacy and safety of an extract of *Pelargonium sidoides* (EPs 7630) in adults with acute bronchitis. A randomised, double-blind, placebo-controlled trial. Phytomedicine 10(4):7–17. 10.1078/1433-187x-00308 12807337 10.1078/1433-187x-00308

[CR19] Mohaddes AA, Saatchi MA, Afshari Chamanabadi M, Saatchi S, Rostami S, Askari VR (2025) Quantum Health Accelerator(®) ameliorates CFA-induced animal model of rheumatoid arthritis: investigating the role of immunomodulatory and anti-oxidative effects. Brain Sci. 10.3390/brainsci15030232 40149754 10.3390/brainsci15030232 PMC11940038

[CR20] Mueller AL, Payandeh Z, Mohammadkhani N, Mubarak SMH, Zakeri A, Alagheband Bahrami A, Brockmueller A, Shakibaei M (2021) Recent advances in understanding the pathogenesis of rheumatoid arthritis: new treatment strategies. Cells. 10.3390/cells10113017 34831240 10.3390/cells10113017 PMC8616543

[CR21] Newbould BB (1963) Chemotherapy of arthritis induced in rats by mycobacterial adjuvant. Br J Pharmacol Chemother 21(1):127–136. 10.1111/j.1476-5381.1963.tb01508.x 14066137 10.1111/j.1476-5381.1963.tb01508.x PMC1703866

[CR22] Nöldner M, Schötz K (2007) Inhibition of lipopolysaccharid-induced sickness behavior by a dry extract from the roots of *Pelargonium sidoides* (EPs 7630) in mice. Phytomedicine 14(Suppl 6):27–31. 10.1016/j.phymed.2006.11.013 17182237 10.1016/j.phymed.2006.11.013

[CR23] Paleolog EM (2002) Angiogenesis in rheumatoid arthritis. Arthritis Res 4(3):S81-90. 10.1186/ar575 12110126 10.1186/ar575 PMC3240151

[CR24] Papapetropoulos A, García-Cardeña G, Madri JA, Sessa WC (1997) Nitric oxide production contributes to the angiogenic properties of vascular endothelial growth factor in human endothelial cells. J Clin Invest 100(12):3131–3139. 10.1172/jci119868 9399960 10.1172/JCI119868 PMC508526

[CR25] Patel R, Kadri S, Gohil P, Deshpande S, Shah G (2021) Amelioration of complete Freund’s adjuvant-induced arthritis by *Calotropis procera* latex in rats. Future J Pharm Sci 7(1):213. 10.1186/s43094-021-00361-w

[CR26] Pearson CM (1956) Development of arthritis, periarthritis and periostitis in rats given adjuvants. Proc Soc Exp Biol Med 91(1):95–101. 10.3181/00379727-91-22179 13297719 10.3181/00379727-91-22179

[CR27] Quiñonez-Flores CM, González-Chávez SA, Del Río Nájera D, Pacheco-Tena C (2016) Oxidative stress relevance in the pathogenesis of the rheumatoid arthritis: a systematic review. BioMed Res Int 2016:6097417. 10.1155/2016/6097417 27340664 10.1155/2016/6097417 PMC4906181

[CR28] Reina BD, Malheiros SS, Vieira SM, Ferreira de Andrade P, Dovigo LN (2024) Unlocking the therapeutic potential of *Pelargonium sidoides* natural extract: a scoping review. Heliyon 10(23):e40554. 10.1016/j.heliyon.2024.e40554 39654721 10.1016/j.heliyon.2024.e40554 PMC11625261

[CR29] Schini-Kerth VB, Auger C, Kim JH, Etienne-Selloum N, Chataigneau T (2010) Nutritional improvement of the endothelial control of vascular tone by polyphenols: role of NO and EDHF. Pflugers Arch 459(6):853–862. 10.1007/s00424-010-0806-4 20224869 10.1007/s00424-010-0806-4

[CR30] Smolen JS, Aletaha D, McInnes IB (2016) Rheumatoid arthritis. Lancet 388(10055):2023–2038. 10.1016/S0140-6736(16)30173-8 27156434 10.1016/S0140-6736(16)30173-8

[CR31] Sukketsiri W, Chonpathompikunlert P, Tanasawet S, Choosri N, Wongtawatchai T (2016) Effects of Apium graveolens extract on the oxidative stress in the liver of adjuvant-induced arthritic rats. Prev Nutr Food Sci 21(2):79–84. 10.3746/pnf.2016.21.2.79 27390722 10.3746/pnf.2016.21.2.79 PMC4935245

[CR32] Tastekin N, Aydogdu N, Dokmeci D, Usta U, Birtane M, Erbas H, Ture M (2007) Protective effects of L-carnitine and alpha-lipoic acid in rats with adjuvant arthritis. Pharmacol Res 56(4):303–310. 10.1016/j.phrs.2007.07.008 17826175 10.1016/j.phrs.2007.07.008

[CR33] Teerlink T, Nijveldt RJ, de Jong S, van Leeuwen PAM (2002) Determination of arginine, asymmetric dimethylarginine, and symmetric dimethylarginine in human plasma and other biological samples by high-performance liquid chromatography. Anal Biochem 303(2):131–137. 10.1006/abio.2001.5575 11950212 10.1006/abio.2001.5575

[CR34] Terlizzi M, Colarusso C, Di Maio U, Bagnulo A, Pinto A, Sorrentino R (2020) Antioxidant and antimicrobial properties of *Pelargonium sidoides* DC and lactoferrin combination. Biosci Rep. 10.1042/bsr20203284 10.1042/BSR20203284 PMC7672805 33119061

[CR35] Ueki Y, Miyake S, Tominaga Y, Eguchi K (1996) Increased nitric oxide levels in patients with rheumatoid arthritis. J Rheumatol 23(2):230–236 8882024

[CR36] Wang Y, Wu H, Deng R (2021) Angiogenesis as a potential treatment strategy for rheumatoid arthritis. Eur J Pharmacol 910:174500. 10.1016/j.ejphar.2021.174500 34509462 10.1016/j.ejphar.2021.174500

[CR37] Zhao F, Hu Z, Li G, Liu M, Huang Q, Ai K, Cai X (2025) Angiogenesis in rheumatoid arthritis: pathological characterization, pathogenic mechanisms, and nano-targeted therapeutic strategies. Bioact Mater 50:603–639. 10.1016/j.bioactmat.2025.04.026 40453697 10.1016/j.bioactmat.2025.04.026 PMC12124647

