# Supplementary material for: Effects of Pelargonium sidoides root extract on paw oedema, nitric oxide signaling, and synovial vascular changes in a rat model of adjuvant-induced arthritis
Source: Inflammopharmacology. 2026 Mar 2;34(4):2567–78. doi: 10.1007/s10787-026-02171-z (PMC13083382; doi:10.1007/s10787-026-02171-z)
Supplement: Supplementary file 1 — Supplementary Material 1. [file 10787_2026_2171_MOESM1_ESM.docx]

**SUPPLEMENTARY MATERIALS**


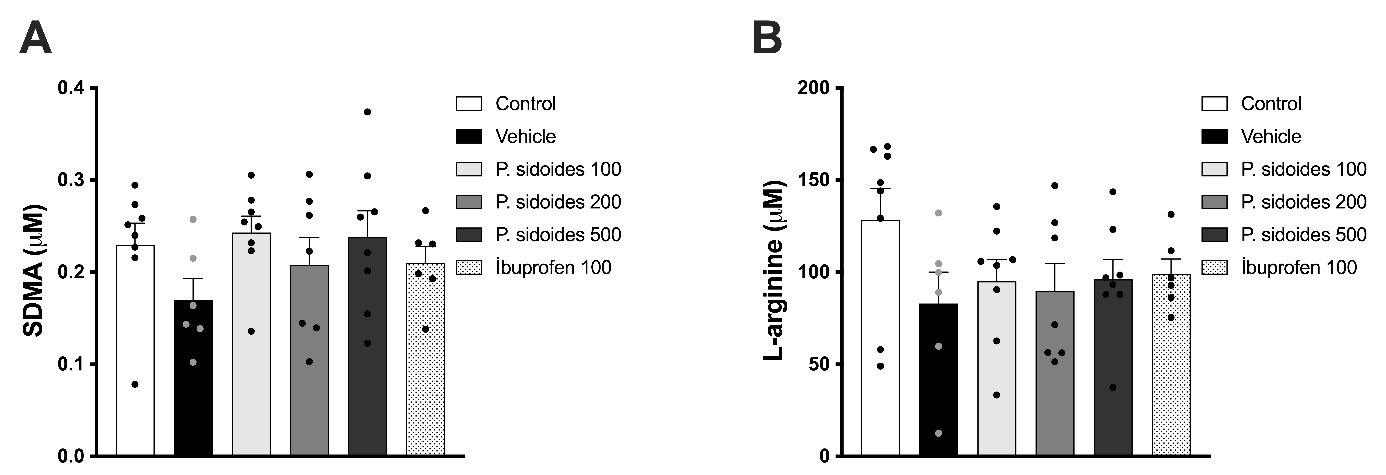


**Supplementary Figure S1. Plasma symmetric dimethylarginine (SDMA) (A) and L-arginine (B) levels across experimental groups.** Data are presented as mean ± SEM (n = 6–8 per group). One-way ANOVA with Tukey’s post hoc test was used.

**
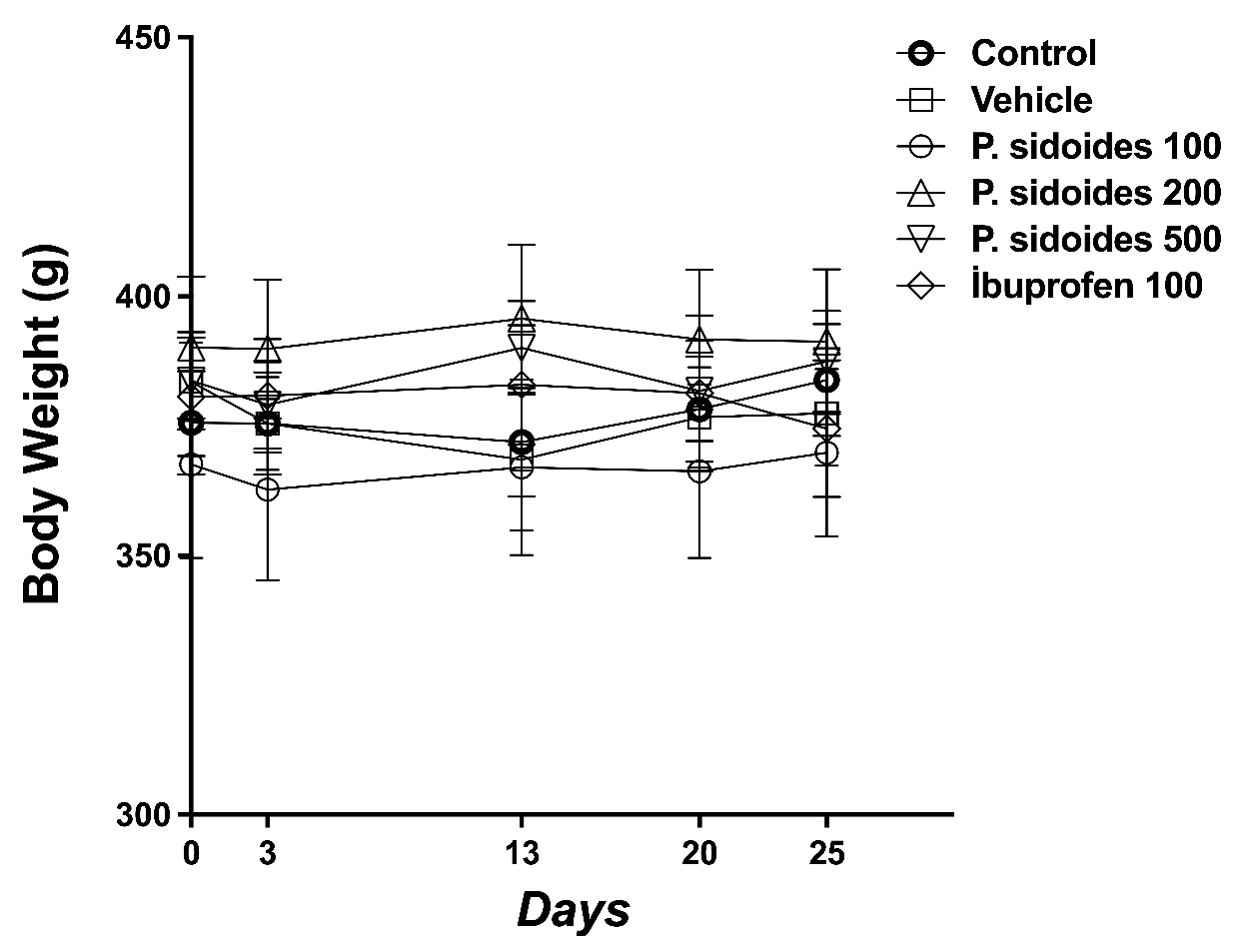
**

**Supplementary Figure S2. Effects of *P. sidoides* treatment on body weight changes in CFA-induced arthritic rats.** Body weight was monitored throughout the study period. No statistically significant weight loss was observed in *P. sidoides*-treated groups compared to the control group. Data are presented as mean ± SEM (n = 6–8 per group).
